# Daily SARS-CoV-2 Nasal Antigen Tests Miss Infected and Presumably Infectious People Due to Viral Load Differences among Specimen Types

**DOI:** 10.1128/spectrum.01295-23

**Published:** 2023-06-14

**Authors:** Alexander Viloria Winnett, Reid Akana, Natasha Shelby, Hannah Davich, Saharai Caldera, Taikun Yamada, John Raymond B. Reyna, Anna E. Romano, Alyssa M. Carter, Mi Kyung Kim, Matt Thomson, Colten Tognazzini, Matthew Feaster, Ying-Ying Goh, Yap Ching Chew, Rustem F. Ismagilov

**Affiliations:** a California Institute of Technology, Pasadena, California, USA; b Pangea Laboratory LLC, Tustin, California, USA; c Zymo Research Corporation, Irvine, California, USA; d Pasadena Public Health Department, Pasadena, California, USA; Johns Hopkins Medicine

**Keywords:** COVID-19, sensitivity, infectious, diagnostics, evaluation

## Abstract

In a recent household transmission study of SARS-CoV-2, we found extreme differences in SARS-CoV-2 viral loads among paired saliva, anterior nares swab (ANS), and oropharyngeal swab specimens collected from the same time point. We hypothesized these differences may hinder low-analytical-sensitivity assays (including antigen rapid diagnostic tests [Ag-RDTs]) by using a single specimen type (e.g., ANS) from reliably detecting infected and infectious individuals. We evaluated daily at-home ANS Ag-RDTs (Quidel QuickVue) in a cross-sectional analysis of 228 individuals and a longitudinal analysis (throughout infection) of 17 individuals enrolled early in the course of infection. Ag-RDT results were compared to reverse transcription-quantitative PCR (RT-qPCR) results and high, presumably infectious viral loads (in each, or any, specimen type). The ANS Ag-RDT correctly detected only 44% of time points from infected individuals on cross-sectional analysis, and this population had an inferred limit of detection of 7.6 × 10^6^ copies/mL. From the longitudinal cohort, daily Ag-RDT clinical sensitivity was very low (<3%) during the early, preinfectious period of the infection. Further, the Ag-RDT detected ≤63% of presumably infectious time points. The poor observed clinical sensitivity of the Ag-RDT was similar to what was predicted based on quantitative ANS viral loads and the inferred limit of detection of the ANS Ag-RDT being evaluated, indicating high-quality self-sampling. Nasal Ag-RDTs, even when used daily, can miss individuals infected with the Omicron variant and even those presumably infectious. Evaluations of Ag-RDTs for detection of infected or infectious individuals should be compared with a composite (multispecimen) infection status to correctly assess performance.

**IMPORTANCE** We reveal three findings from a longitudinal study of daily nasal antigen rapid diagnostic test (Ag-RDT) evaluated against SARS-CoV-2 viral load quantification in three specimen types (saliva, nasal swab, and throat swab) in participants enrolled at the incidence of infection. First, the evaluated Ag-RDT showed low (44%) clinical sensitivity for detecting infected persons at all infection stages. Second, the Ag-RDT poorly detected (≤63%) time points that participants had high and presumably infectious viral loads in at least one specimen type. This poor clinical sensitivity to detect infectious individuals is inconsistent with the commonly held view that daily Ag-RDTs have near-perfect detection of infectious individuals. Third, use of a combination nasal-throat specimen type was inferred by viral loads to significantly improve Ag-RDT performance to detect infectious individuals.

## INTRODUCTION

Antigen rapid diagnostic tests (Ag-RDTs) with nasal swabs are increasingly used for SARS-CoV-2 screening and diagnosis globally (1–3). Ag-RDTs are powerful tools given their low cost (compared with molecular tests), speed, and portability, making them appropriate for low-resource settings and at-home use (2, 4, 5). However, Ag-RDTs and some rapid molecular tests have lower analytical sensitivity than most gold-standard reverse transcription-quantitative PCR (RT-qPCR) tests and therefore require high viral loads (typically >10^5^ copies/mL) to reliably yield positive results (4, 6–11). Some contend that Ag-RDTs may miss some infected individuals but will result as positive when individuals are infectious with high viral loads (12–14). Such concordance would allow high-frequency Ag-RDTs (with immediate results) to more effectively prompt isolation of infectious individuals than a high-analytical-sensitivity test (with delayed results) (12, 15).

Investigating Ag-RDT performance for detecting the infectious period by viral culture is challenging and infrequently performed. Instead, because replication-competent virus is associated with viral loads ≥10^4^ copies/mL in studies that have performed SARS-CoV-2 viral culture (see Table S1 in the supplemental material), viral load is often used as a surrogate for infectiousness. Longitudinal studies that captured viral load measurements from early in infection (16–30) show that for some individuals, several days can pass between when viral loads reach potentially infectious levels and when viral loads rise to the limits of detection (LODs) of Ag-RDTs (~10^5^ to 10^7^ copies/mL) (4, 6–10, 20, 21, 31). During this window, false-negative Ag-RDT results may occur, emboldening social contact and increasing transmission (32, 33).

In our household transmission study analyzing viral loads from daily sampling of anterior nares nasal swabs (ANS), oropharyngeal swabs (OPS), and saliva (SA) beginning from the incidence of SARS-CoV-2 Omicron infection, two findings suggested Ag-RDTs may miss many infected and infectious individuals (26). First, viral loads for an individual often differed significantly (>9 orders of magnitude) among specimen types at the same time point and did not correlate with each other over time. Individuals often had high, presumably infectious viral loads in one type (e.g., OPS), yet low loads in another (e.g., ANS). Because all at-home Ag-RDTs authorized by the U.S. Food and Drug Administration (FDA) are for nasal swabs (7), this lack of correlation among specimen types could hinder the ability of Ag-RDTs to detect infectious individuals with high loads in nonnasal specimen types. Second, we observed that most individuals exhibit a delay in the rise of ANS viral loads relative to the oral cavity (26); this finding is consistent with previous reports by us (21) for ancestral SARS-CoV-2 variants and other studies (17, 18, 20, 25) that included the early period of infection in multiple specimen types. A delayed rise in ANS viral loads could delay nasal Ag-RDT detection of infected and infectious individuals.

These underlying viral load patterns impact interpretation of Ag-RDT field evaluations. Although many Ag-RDT evaluations report concordance with infectiousness (by viral culture [16–19, 34–44] or presumed by quantitative viral loads or semiquantitative threshold cycle [*C_T_*] values [45–48]), in several studies (16, 34, 36, 37, 41, 43, 45, 47, 48), most participants were already symptomatic, so results may not generalize to early infection. Among longitudinal nasal Ag-RDT studies that accounted for infection stage (17–19, 35, 38, 39, 43), some (17–19, 35, 38) used prospective sampling to capture early infections, but none tested for infectious virus in multiple specimen types. To our knowledge, only one nasal Ag-RDT evaluation examined infectiousness in oral specimens; the Ag-RDT was often negative while individuals had infectious loads in saliva (20). There is a paucity of data on Ag-RDT performance in early infection and compared to infectiousness in multiple upper respiratory specimen types.

**FIG 1:**
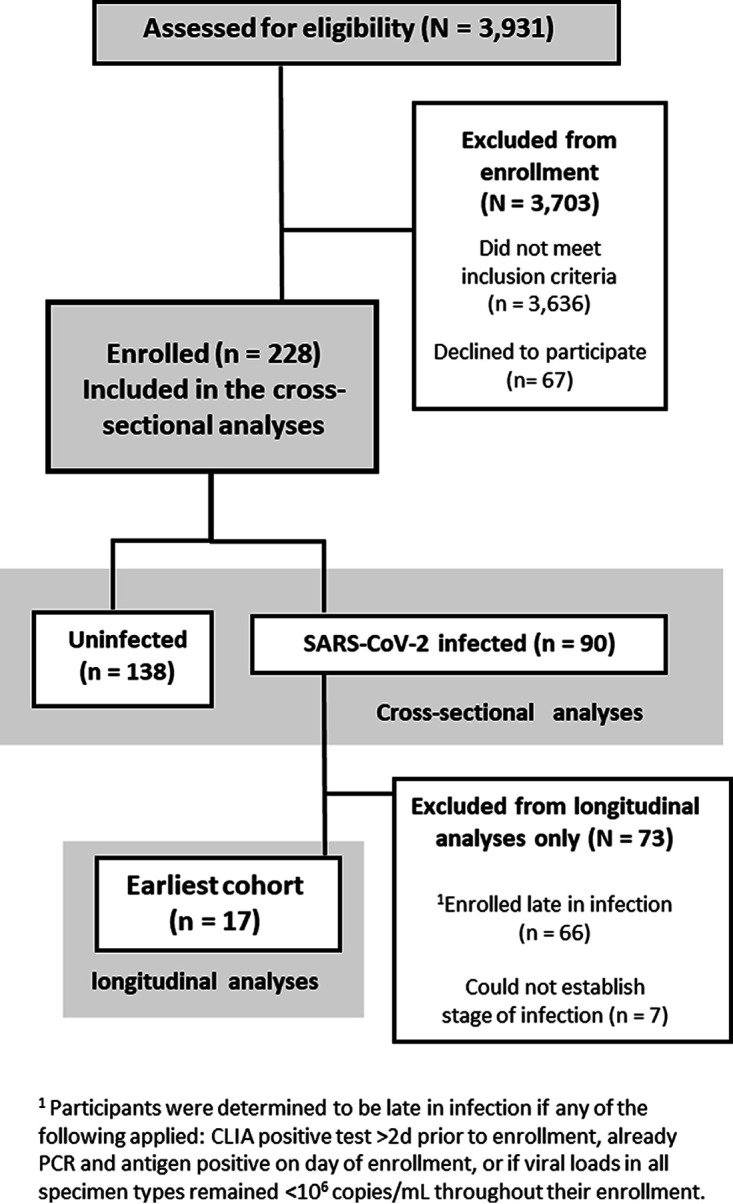
CONSORT diagram of participant recruitment, eligibility, and enrollment for the cross-sectional and longitudinal analyses (adapted from reference 26). Demographic and medical information can be found in Tables S1 and S2 in the supplemental material. Cross-sectional analyses are presented in Fig. 2 and 3; longitudinal analyses are presented in Fig. 4 to 6.

Here, we report a field evaluation of an ANS Ag-RDT (QuickVue At-Home OTC COVID-19 test), with cross-sectional and longitudinal analyses (Fig. 1). A daily ANS Ag-RDT was taken prospectively by participants with a recently infected or exposed household contact. Participants also collected daily SA, ANS, and OPS specimens for SARS-CoV-2 testing and viral load quantification (26). From these viral load measurements, we assessed Ag-RDT performance to identify individuals with detectable or presumably infectious viral loads in any of the three specimen types. This design allowed us to probe the performance of this Ag-RDT for early detection and identify underlying reasons why Ag-RDTs may exhibit poor performance to detect infected and infectious individuals.

## RESULTS

### Ag-RDT detects <50% of infected individuals.

**FIG 2:**
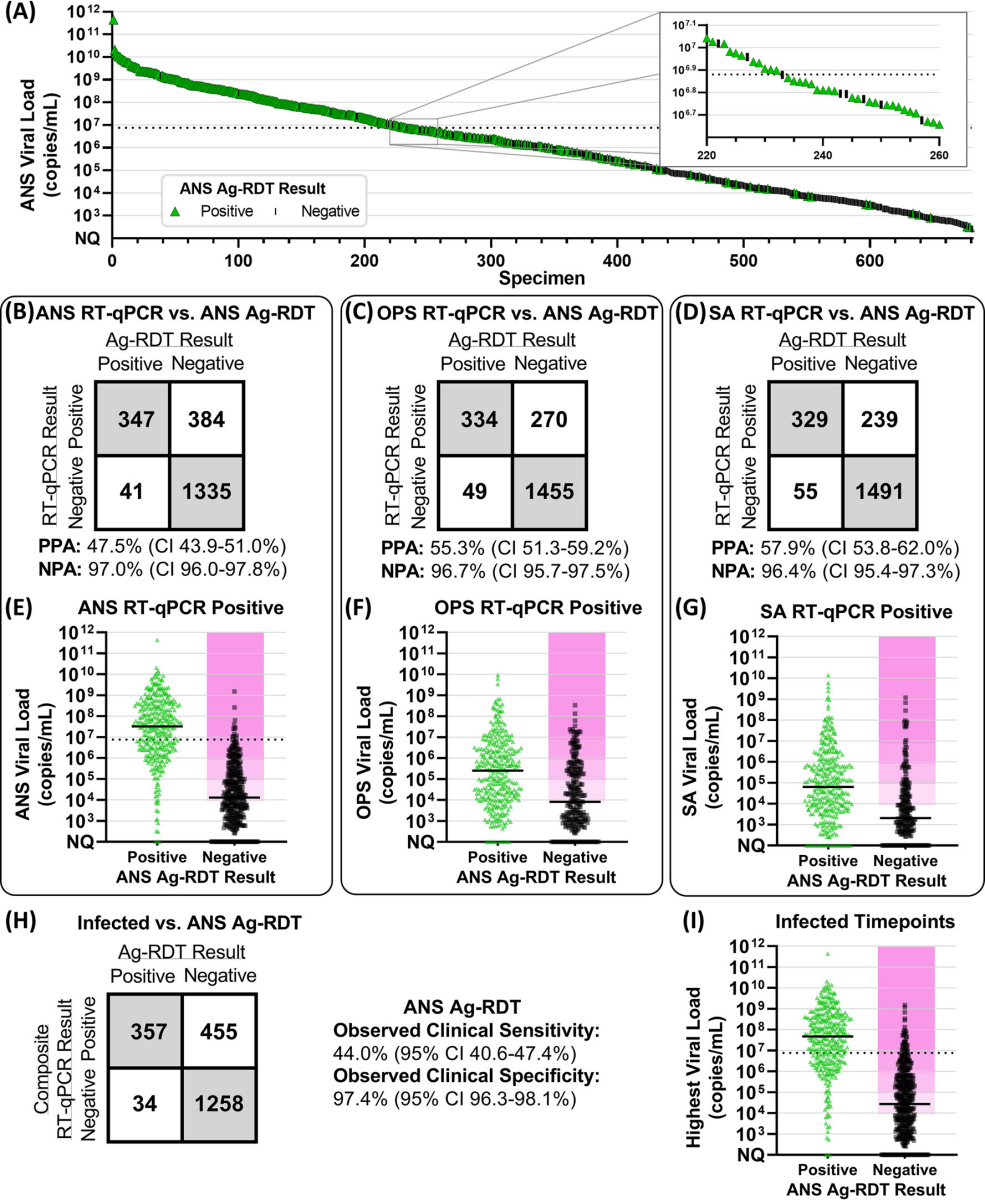
Comparison of anterior nares swab antigen rapid diagnostic test (Ag-RDT) results to RT-qPCR results and viral loads. (A) We ordered 680 ANS specimens with quantifiable SARS-CoV-2 viral loads by viral load and colored them by Ag-RDT results (green for positive antigen test result, black for antigen negative). Inset shows higher resolution for results with viral loads around 7.6 × 10^6^ copies/mL (black dashed line), above which 95% of ANS specimens resulted Ag-RDT positive. (B to D) Two by two matrices of concordance between ANS Ag-RDT results and valid, conclusive RT-qPCR results for 2,107 ANS specimens (B), 2,108 OPS specimens (C), and 2,114 SA specimens (D). PPA, positive percent agreement; NPA, negative percent agreement. CI indicates 95% confidence interval. (E to G) Distribution of viral loads from 731 RT-qPCR-positive ANS specimens (E), 604 RT-qPCR-positive OPS specimens (F), and 568 RT-qPCR-positive SA specimens (G), with either positive or negative Ag-RDT results. Solid horizontal black lines indicate medians. (H) Two by two matrix of observed concordance between Ag-RDT results and infected status, based on composite RT-qPCR results from all three specimen types, at 2,104 time points, with valid, conclusive results for all specimen types by RT-qPCR and valid ANS Ag-RDT results. (I) Distribution of the highest viral load among ANS, OPS, and SA specimens collected by any participant at 812 composite RT-qPCR-positive (infected) time points, with either positive or negative Ag-RDT results. Magenta shading in panels E, F, G, and I indicates infectious viral loads (above 10^4^, 10^5^, 10^6^, or 10^7^ copies/mL). ANS, anterior nares swab; OPS, oropharyngeal swab; SA, saliva; Ag-RDT, antigen rapid diagnostic test. Detailed tabulation, including inconclusive and invalid results, is shown in Table S2 in the supplemental material.

We first performed a cross-sectional analysis to estimate the LOD of the ANS Ag-RDT and then compared the positive percent agreement (PPA) of the ANS Ag-RDT against ANS RT-qPCR or composite infection status (based on RT-qPCR results from ANS, OPS, and SA). Of 680 ANS specimens with quantifiable viral loads and valid, paired ANS Ag-RDT results, 95% PPA was observed when ANS specimens had viral loads ≥7.6 × 10^6^ copies/mL (Fig. 2A), suggesting this value as an inferred estimate of the assay LOD (16, 31, 49). We observed 48% (347 of 731) PPA between the ANS Ag-RDT and ANS RT-qPCR (Fig. 2B). However, the observed clinical sensitivity of the ANS Ag-RDT (Fig. 2H) compared to composite infection status was 44% (357 of 812 infected time points), significantly lower (*P < *0.001, upper-tailed McNemar exact test) than the PPA against ANS RT-qPCR alone (Fig. 2B). Although low PPA and clinical sensitivity to detect infection were expected due to the low analytical sensitivity of the Ag-RDT, the Ag-RDT resulted negative at many time points that participants had high, presumably infectious viral loads in ANS, SA, or OPS specimens (Fig. 2E to G). Approximately 50% of time points at which the ANS Ag-RDT resulted negative had viral loads above 10^4^ copies/mL in ANS (Fig. 2E), OPS (Fig. 2F), or any specimen type (Fig. 2I).

### Analytical sensitivity, IVLT, and specimen type strongly impact the ability to detect infectious individuals.

**FIG 3:**
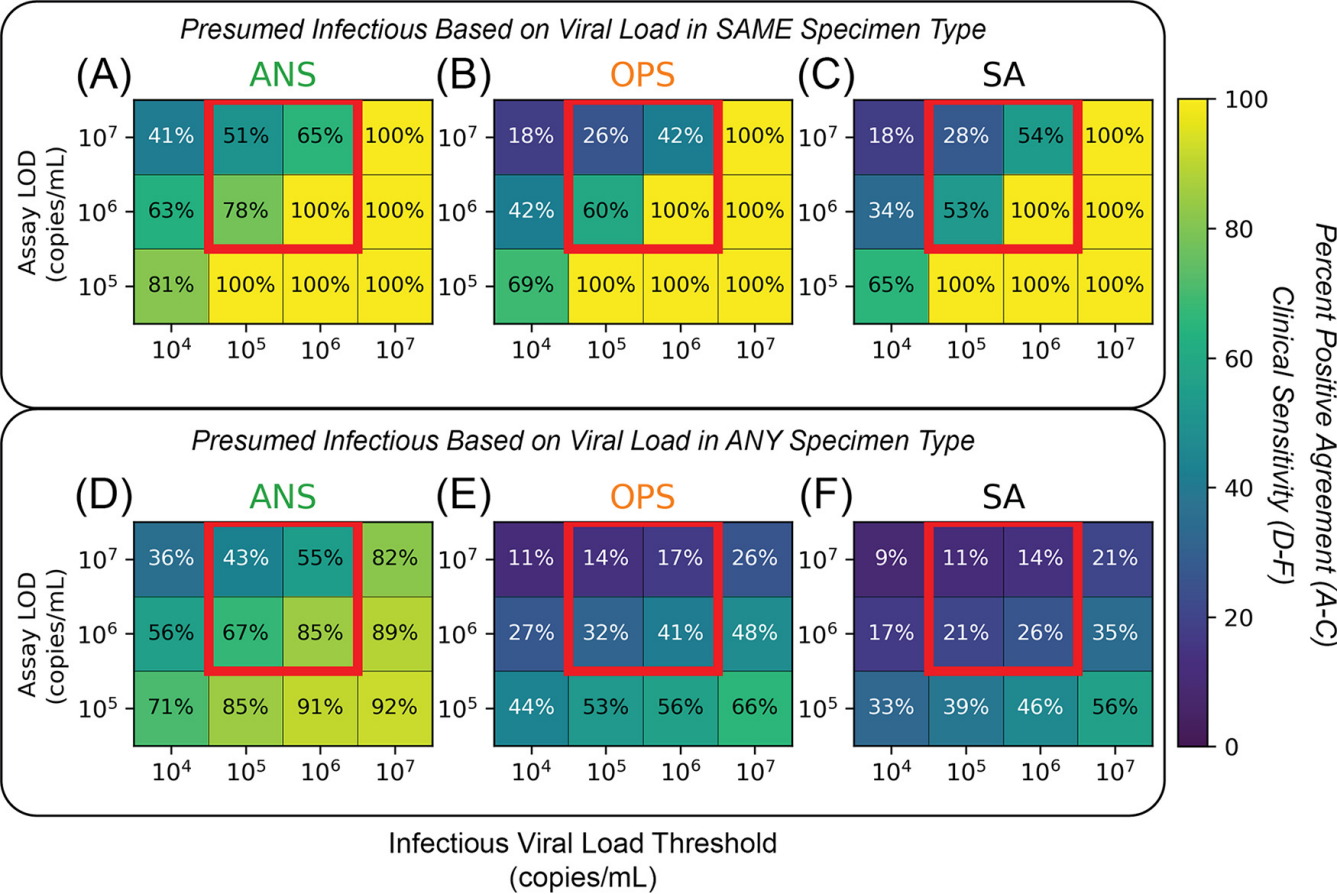
Effects of low-analytical-sensitivity assay LOD, infectious viral load threshold (IVLT), and inclusion of multiple specimen types on inferred clinical sensitivity to detect presumed infectious individuals. (A to C) Heatmaps visualizing positive percent agreement for each specimen type, showing anterior nares swab (ANS) (A), oropharyngeal swab (OPS) (B), and saliva (SA) (C), tested with assays of different LODs in the range of low-analytical-sensitivity tests (such as Ag-RDTs) to detect individuals presumed infectious only if the viral load in the tested specimen type was at or above a given IVLT. Red boxes highlight an important interaction between assay LOD and IVLT that is elaborated in the text. (D to F) Heatmaps visualizing the inferred clinical sensitivity for each specimen type, including ANS (A), OPS (B), and SA (C), tested with assays of different LODs to detect individuals presumed to be infectious if the viral load in any specimen type was at or above a given IVLT. Heatmaps for computationally contrived combination specimen types are shown in Fig. S2 in the supplemental material.

We next assessed how well presumably infectious individuals would be detected by low-analytical-sensitivity assays. Infectious viral load thresholds (IVLTs) were used to classify individuals as infectious. To examine differences resulting from IVLT selection, we created a matrix of IVLTs (10^4^, 10^5^, 10^6^, or 10^7^ copies/mL) and low-analytical-sensitivity assay LODs (10^5^ to 10^7^ copies/mL) for each specimen type. In each cell, we calculated inferred clinical sensitivity for each hypothetical assay to detect presumed infectious time points. We calculated inferred clinical sensitivity against time points with viral loads above the IVLT only in one specimen type (Fig. 3A to C) and against time points with a viral load above the IVLT in any of the three specimen types (Fig. 3D to F).

When considering viral loads only in the specimen type tested, clinical sensitivity increased as IVLT increased and decreased as LOD increased. Setting an IVLT at or above the LOD of the assay artificially increased the inferred clinical sensitivity to detect presumed infectious individuals. We highlight three instances (red boxes in Fig. 3A to C) where inferred clinical sensitivities increased by up to 74% as a result of IVLT selection. Perfect performance was observed where IVLT was at or above the assay LOD (lower right cells in Fig. 3A to C). This analysis demonstrates how selection of an IVLT similar to the assay LOD will overestimate clinical sensitivity to detect infectious individuals.

Importantly, when considering viral loads above the IVLT in any of the three specimen types tested (Fig. 3D to F), inferred clinical sensitivities were lower for all specimen types, regardless of IVLT or assay LOD. Because of extreme differences in viral load among specimen types from the same individual at a given time point (26), individuals often had high, presumably infectious viral loads in one, but not all, specimen types. Thus, inferred clinical sensitivity decreased drastically when infectiousness in multiple specimen types, rather than just one, is considered.

### Longitudinal Ag-RDT performance.

**FIG 4:**
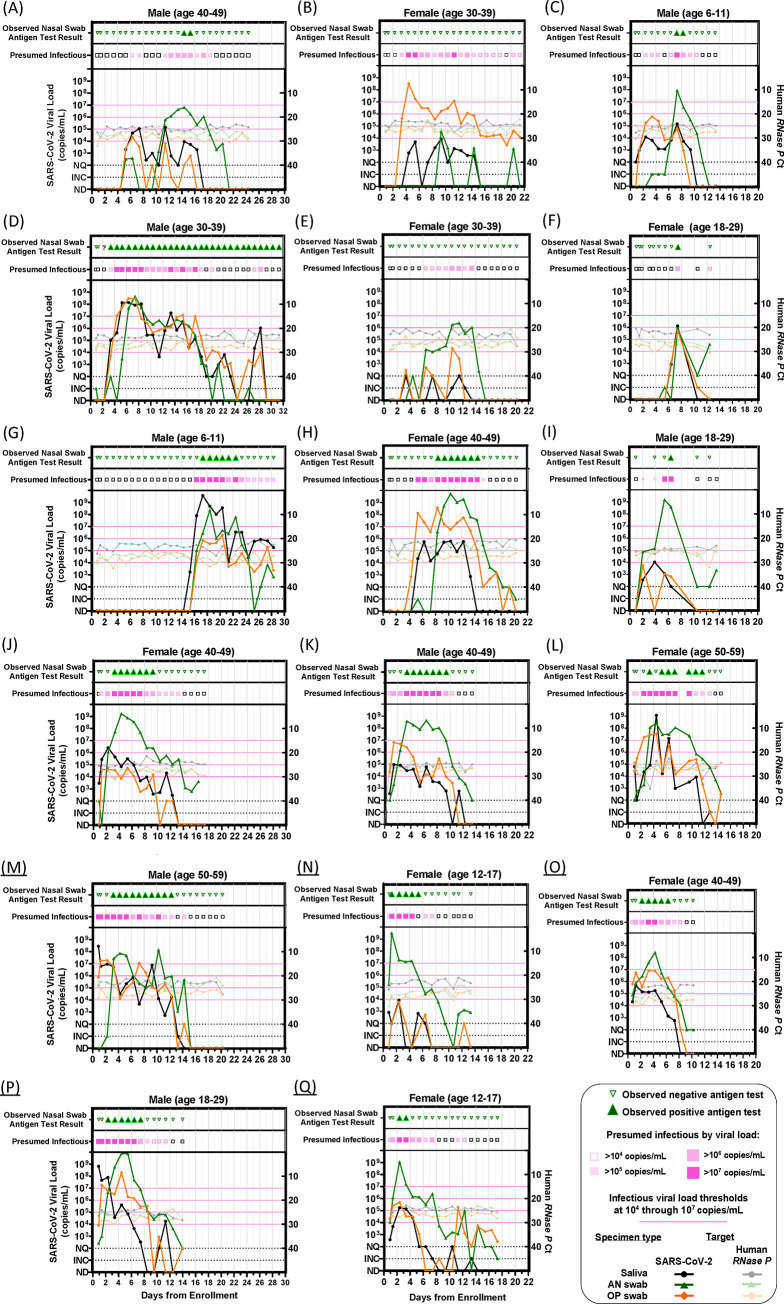
Longitudinal viral loads and antigen rapid diagnostic testing. Each panel (A to Q) represents a single participant throughout the course of enrollment, with observed ANS rapid antigen testing results, presumed infectious period (magenta) based on viral loads at or above each infectious viral load threshold 10^4^ to 10^7^ copies/mL in any specimen type, SARS-CoV-2 viral loads (left *y* axis), and human RNase P *C_T_* values (right *y* axis) by RT-qPCR in each specimen type. INC, inconclusive; NQ, viral load detected but below the test LOD (250 copies/mL); ND, not detected for RT-qPCR measurements; AN, anterior nares; OP, oropharyngeal. A single invalid antigen test is indicated with a “?” symbol in panel D. (Panels A to N were adapted from reference 26, in which viral load data for participants A to N were reported previously).

We next assessed the performance of the ANS Ag-RDT longitudinally through acute infection. We identified a cohort of 17 individuals who began sampling early in the course of infection (Fig. 1). We compiled participants’ daily viral load measurements for each specimen type (SA, ANS, OPS) (26) with paired ANS Ag-RDT results and classified time points as presumably infectious when the viral load in any of the three specimen types was above a given IVLT (Fig. 4).

All but two of the 17 participants (Fig. 4D and F) reached presumed infectious viral loads at least 1 day before their first positive Ag-RDT result. Of these 15 participants, 6 had a delay of 1 to 2 days (Fig. 4G, J, and N to Q), 5 had a delay of 3 days (Fig. 4H, I, and K to M), 1 had a delay of 5 days (Fig. 4C), and 1 had a delay of 8 days (Fig. 4A). Two participants (Fig. 4B and E) had infectious viral loads for more than 8 days each, but neither ever reported a positive ANS Ag-RDT result. The participant in Fig. 4B had high (>10^5^ copies/mL) OPS viral loads for 12 days, while ANS specimens remained low (rising just above 10^4^ copies/mL only once). The participant in Fig. 4E had ANS viral loads >10^6^ copies/mL on 3 days but never yielded a positive Ag-RDT result, likely because these viral loads were near the Ag-RDT LOD.

In this cohort, the overall observed clinical sensitivity of the ANS Ag-RDT to detect infected individuals was significantly higher when participants were symptomatic (Fig. S1), but low (<50%) at both symptomatic and asymptomatic time points.

### Nasal Ag-RDT misses infectious viral loads in other specimen types.

**FIG 5:**
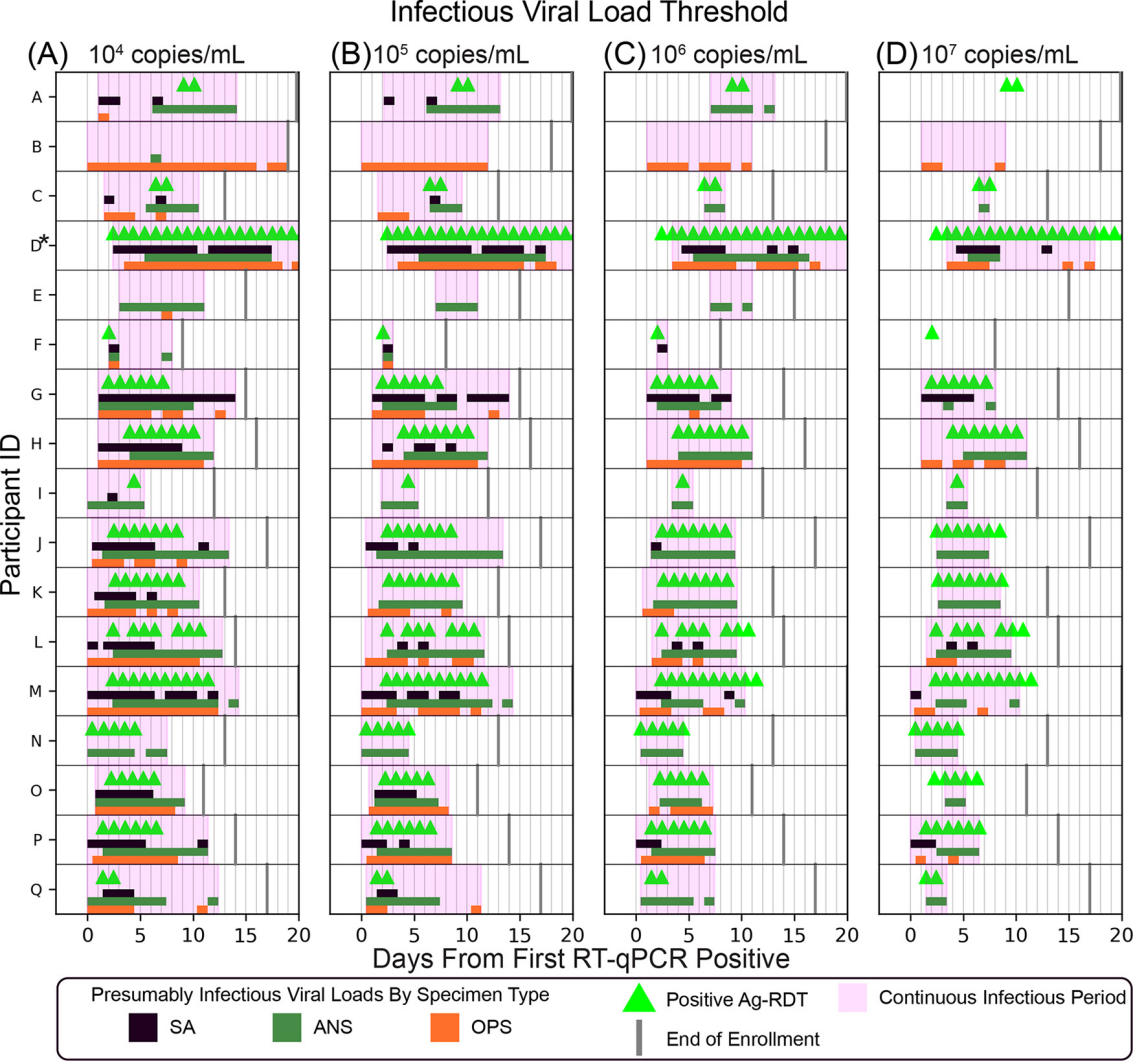
Periods of presumed infectiousness as a factor of infectious viral load threshold (IVLT). (A to D) Days starting from first RT-qPCR positive that each participant (A to Q; see Fig. 3) had presumably infectious viral loads (with IVLTs of 10^4^ to 10^7^ copies/mL) in each specimen type (green bars, anterior nares swab [ANS]; orange bars, oropharyngeal swab [OPS]; black bars, saliva [SA]). Positive Ag-RDT tests are indicated with green triangles, and the final date of study enrollment for each is indicated with gray lines. The time course for participant D (who experienced a series of false-positive antigen tests) is truncated, indicated by an asterisk (see supplemental information).

Given that many individuals had high, presumably infectious viral loads before their first ANS Ag-RDT-positive result (Fig. 4), we next assessed how periods of infectiousness in each of the three specimen types overlapped and which time points were detected by the ANS Ag-RDT. We aligned each participant’s time course to their first RT-qPCR-positive result in any specimen type and then plotted the period each specimen type had viral loads above the IVLT. Periods when viral loads were above the IVLT in any specimen type are indicated in magenta. Positive ANS Ag-RDT results were overlaid (Fig. 5).

For IVLTs below 10^7^ copies/mL (Fig. 5A to C), all 17 individuals were presumably infectious for at least 1 day. As IVLT increased, the length of the infectious period for each participant decreased. At an IVLT of 10^7^ copies/mL (Fig. 5D), three participants (participants A, E, and F) would not be considered infectious.

If infectious periods in OPS and SA overlapped perfectly with infectious period in ANS, then OPS and SA viral loads would not affect the performance of the ANS Ag-RDT to detect infectious individuals. However, this was not the case. The presumed infectious periods for different specimen types were often asynchronous (nonoverlapping). For many individuals, OPS or SA specimens reached infectious viral loads prior to ANS. Thus, the Ag-RDT often resulted negative during the infectious period (pink-shaded days lacking green triangles in Fig. 5), particularly in the first days of the infectious period.

### Performance of Ag-RDT in preinfectious and infectious periods.

We next investigated the performance of the daily ANS Ag-RDT to detect individuals during the preinfectious and infectious periods. For each IVLT, the observed clinical sensitivity of the Ag-RDT was plotted alongside inferred clinical sensitivity predicted for ANS specimens tested by a hypothetical assay with a similar LOD (10^6^ copies/mL).

**FIG 6:**
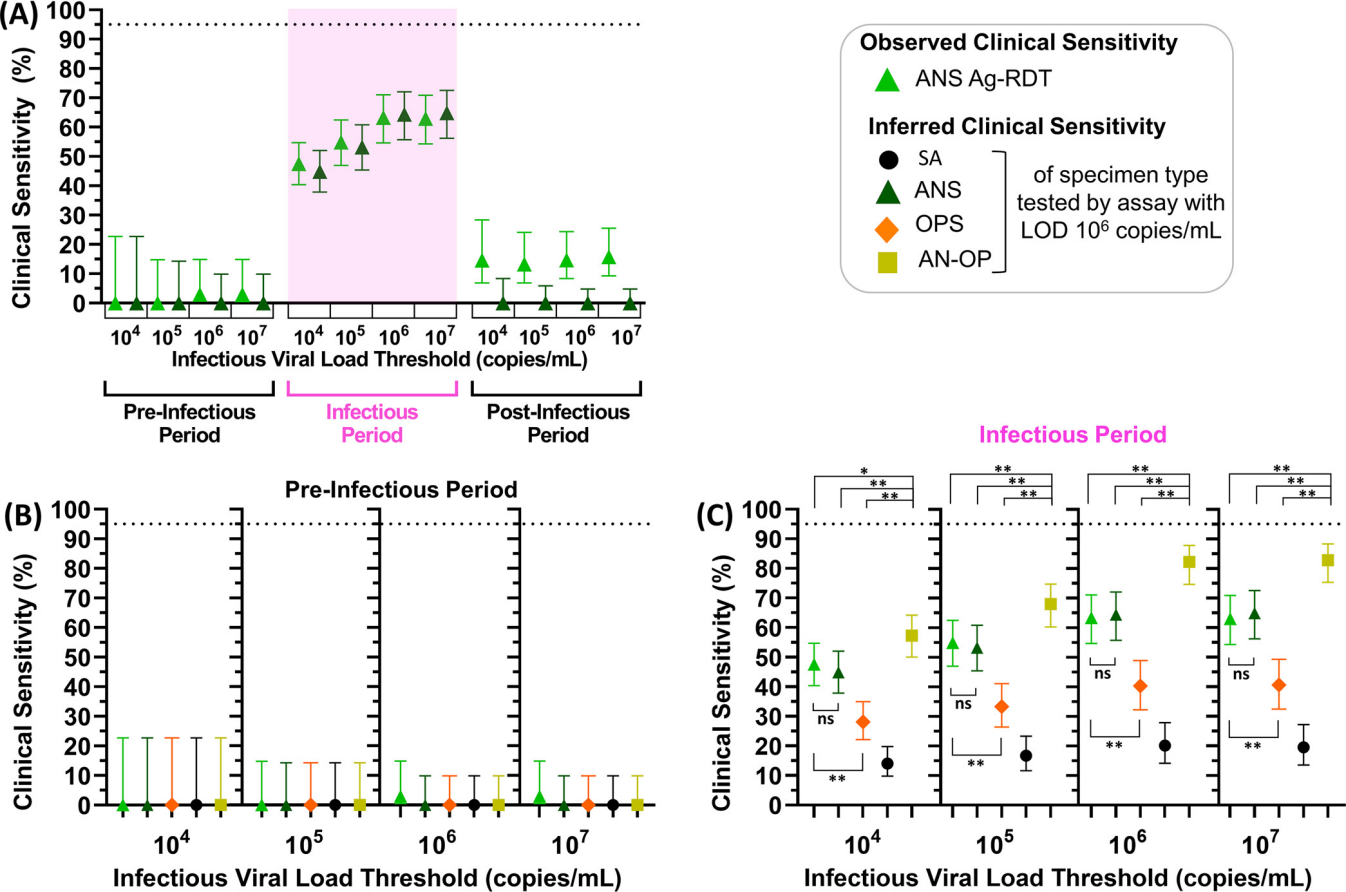
Observed and inferred performance of low-analytical-sensitivity daily antigen rapid diagnostic tests (Ag-RDTs) to detect presumed infectious individuals. Individuals were presumed infectious for the period between first specimen (of any type) with a viral load above the infectious viral load threshold (10^4^, 10^5^, 10^6^, or 10^7^ copies/mL) until all specimen types were below the IVLT; specimens collected prior to this period were considered preinfectious and, after this period, postinfectious. (A) Observed clinical sensitivity of the ANS Ag-RDT (fluorescent green) and the inferred clinical sensitivity of an ANS test with an LOD of 10^6^ copies/mL (green) for each stage of infection. (B and C) Subsequent plots show the observed clinical sensitivity for detection of presumed infectious individuals by the ANS Ag-RDT (fluorescent green) and the inferred clinical sensitivity for ANS (green), OPS (orange), SA (black), and a computationally contrived AN-OP combination swab specimen type (yellow) during the preinfectious period (B) and infectious period (C) of infection. Inferred clinical sensitivity was based on measured viral loads in the given specimen type at or above an LOD of 10^6^. Error bars indicate 95% confidence intervals. Comparison of the clinical sensitivities to detect infectiousness at IVLTs of 10^4^ to 10^7^ across specimen types was performed using the McNemar exact test for given comparisons across specimen type. ANS Ag-RDT versus ANS with LOD 10^6^ copies/mL was tested using a two-tailed McNemar exact test; all other combinations use a one-tailed McNemar exact test. *P* values were adjusted using a Benjamini-Yekutieli correction to account for multiple hypotheses being tested. *, *P* < 0.05; **, *P* < 0.01; ns, *P* ≥ 0.05. Point estimates for these comparisons are provided in Table S4 in the supplemental material. Additional analyses of the inferred clinical sensitivity of the anterior nares-oropharyngeal combination swab can be found in a separate manuscript (26). SA, saliva; ANS, anterior nares swab; OPS, oropharyngeal swab; AN-OP, anterior nares-oropharyngeal combination swab; LOD, limit of detection.

The inferred clinical sensitivity predicted for ANS specimens tested by this hypothetical assay and the observed clinical sensitivity of the Ag-RDT were similar for both the preinfectious and infectious periods at all four IVLTs (Fig. 6A). This congruency supported the use of quantitative viral loads to predict Ag-RDT performance. In the preinfectious period, the Ag-RDT was positive in, at most, 1 of 34 time points (Fig. 6B). In the infectious period, the Ag-RDT detected only 63% of presumed infectious individuals in the highest IVLT (10^7^ copies/mL) (Fig. 6C). Performance decreased as IVLT was lowered; at an IVLT of 10^4^ copies/mL, the Ag-RDT detected only 48% of infectious individuals.

We also inferred the clinical sensitivity of other specimen types if tested by an assay with similar analytical sensitivity as the Ag-RDT. At an LOD of 10^6^ copies/mL, no single specimen type (ANS, OPS, SA) achieved 95% inferred clinical sensitivity to detect infectious individuals for any IVLT (Fig. 6C). However, a computationally contrived AN-OP combination swab specimen at an LOD of 10^6^ copies/mL was predicted to perform significantly better than all other specimen types, including the observed performance of the ANS Ag-RDT (Fig. 6D, Table S4). But, at this low analytical sensitivity, the AN-OP swab was unable to detect preinfectious time points.

## DISCUSSION

Our field evaluation of an ANS Ag-RDT revealed three key findings generally relevant to the use of Ag-RDTs and other tests with low and moderate analytical sensitivity (including some molecular tests that forgo nucleic acid extraction and purification). First, the evaluated Ag-RDT showed low (44%) clinical sensitivity for detecting infected persons at any stage of infection. This poor clinical sensitivity is consistent with another field evaluation of this Ag-RDT used for twice-weekly screening testing at a college (50) It is also consistent with FDA (51) and CDC guidance (52) that using two or more repeat ANS Ag-RDTs are needed to improve the clinical sensitivity of these tests.

**FIG 7:**
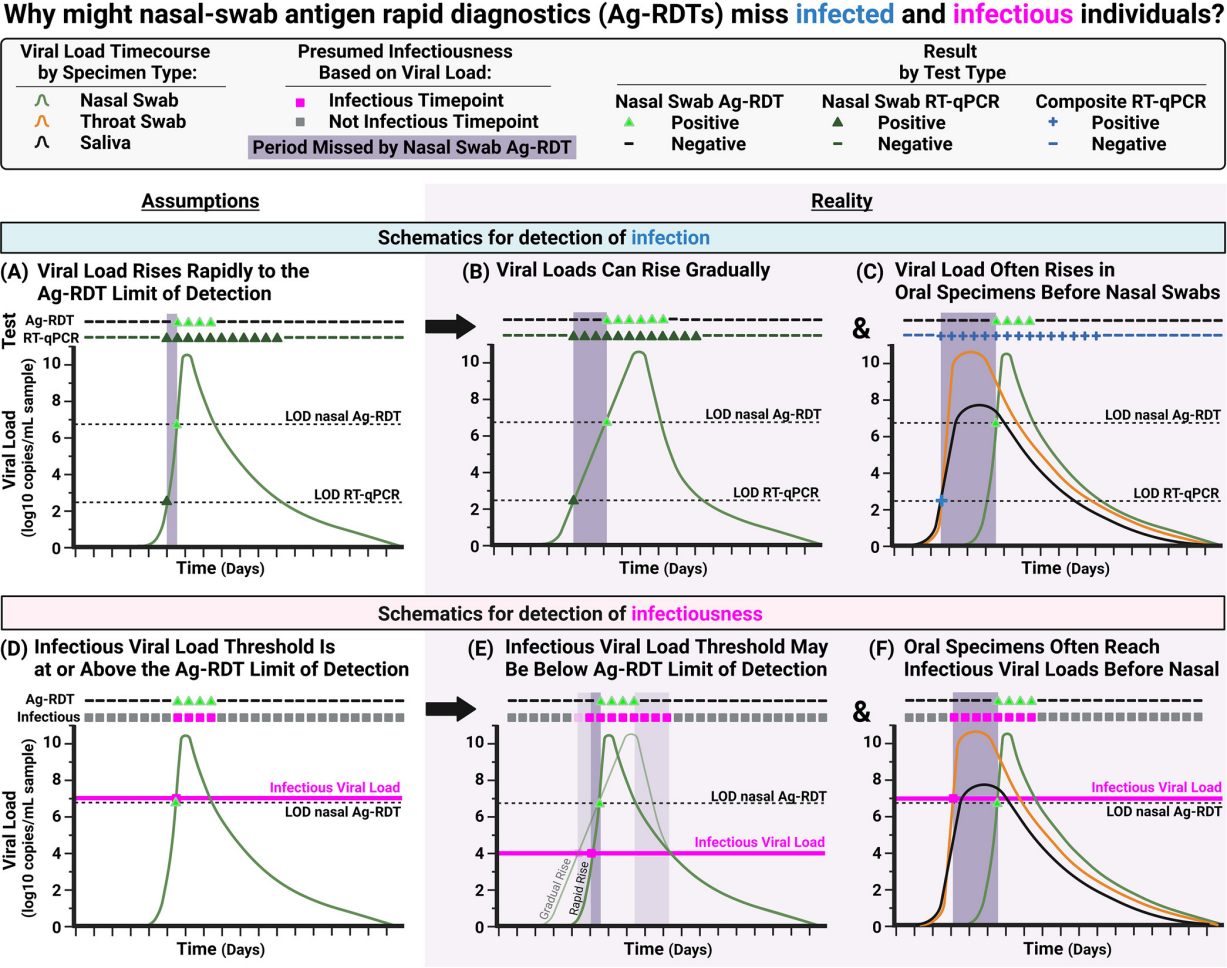
Conceptual diagrams illustrating why nasal-only antigen rapid diagnostic tests (Ag-RDTs) are likely to miss infected and infectious individuals. (A) Schematic of an idealized, hypothetical viral load time course in which viral load rises quickly from detectable to the limit of detection (LOD) of a low-analytical-sensitivity test, such as Ag-RDTs. Such a pattern would result in a daily Ag-RDT being effective for detection of infection (diagram based on a commonly held view [12]). (B) Schematic of a viral load time course based on longitudinal viral load data (16–30) in which, for some individuals, early viral loads rise gradually, resulting in detection of the infected individual several days earlier by a high-analytical-sensitivity test than by the Ag-RDT. This mechanism for missed detection by COVID-19 Ag-RDTs has been previously hypothesized (62). (C) Schematic of a viral load time course based on observed paired longitudinal viral load data in which individuals exhibit a rise in viral load in oral (saliva or throat swab) specimens days before viral loads rise in nasal specimens (17, 18, 20, 21, 25, 26). When these additional specimen types are used to assign a composite infection status, the nasal Ag-RDT is revealed to have poor performance. (D) Schematic of an idealized, hypothetical viral load time course (based on commonly held views [12, 16, 63]) in which viral load rises quickly from detectable to infectious, and the infectious viral load threshold is equivalent to the LOD of the Ag-RDT. Such a pattern would result in near-perfect detection of infectious individuals by the daily Ag-RDT. (E) Schematic of a viral load time course in which the infectious viral load is lower than the LOD of the Ag-RDT. Here, infectious individuals would be missed by the Ag-RDT during the period that the viral load is between the infectious viral load threshold and the LOD of the Ag-RDT. This period will be longer if the rise in viral load is gradual (light-green line) rather than quick (dark-green line). (F) Schematic of a viral load time course in which individuals exhibit high, presumably infectious viral loads in saliva or throat swab specimens while nasal swab viral loads remain very low, particularly at the beginning of infection. Here, the ANS Ag-RDT is unable to detect most infectious time points. The dashed LOD nasal Ag-RDT line indicates the inferred LOD for the nasal-swab Ag-RDT we evaluated (7.6 × 10^6^ copies/mL). The dashed LOD RT-qPCR line indicates the LOD of the RT-qPCR assay used in this study (250 copies/mL). The pink infectious viral load line indicates a threshold associated with the presence of replication-competent virus; individuals are considered infectious if any specimen type has a viral load above the threshold.

There are two reasons for the observed low clinical sensitivity of the ANS Ag-RDT to detect infected individuals. (i) First, the low analytical sensitivity of Ag-RDTs requires high viral loads to yield a positive result. Although it has been proposed (12) that a rapid rise in viral load reduces the advantage of tests that can detect low viral loads (Fig. 7A), this advantage remains when there is a more gradual rise in viral loads (Fig. 7B) as we observed in some individuals (Fig. 4A and C). (ii) The second, more impactful reason is that many early infection time points had detectable virus in saliva or throat swabs, but not ANS. A nasal swab reference test would miss these infected time points. Therefore, the true performance of an ANS Ag-RDT would be worse than composite infection status based on multiple specimen types than nasal swab alone (Fig. 7C).

These two reasons for poor detection of infected individuals by Ag-RDTs have implications for the design and interpretation of other Ag-RDT evaluations. Because viral load time courses in different specimen types from an individual are asynchronous (26), the true clinical sensitivity of an Ag-RDT will be lower than reported by field evaluations that compare only to an ANS reference test (19, 39, 42–44, 46–48, 53). The PPA reported by the Ag-RDT manufacturer to the FDA (83.5%) was calculated relative to detection by a nasal RT-PCR reference test, and nearly all specimens (84 of 91) were from symptomatic individuals likely late in infection (49). Our work suggests that governing bodies should require clinical sensitivity estimates for an Ag-RDT to detect infected individuals to be based on a composite infection status from multiple upper respiratory specimen types.

Our second key finding is that the Ag-RDT poorly detected presumably infectious individuals. The Ag-RDT detected ≤63% of presumed infectious time points. This low clinical sensitivity to detect infectious individuals is inconsistent with a common view (12) that proposes low-analytical-sensitivity tests have near-perfect detection of infectious individuals (Fig. 7D).

Our data demonstrate that this common but idealized view misses two important points. (i) First, in the common view, the LOD of the Ag-RDT aligns with the IVLT (Fig. 7D), but there is no fundamental reason why the LOD should align perfectly with the IVLT. Replication-competent virus is reliably isolated from specimens with viral loads of ≥10^4^ copies/mL (see Table S1 in the supplemental material), whereas Ag-RDT LODs span orders of magnitude (~10^5^ to 10^7^ copies/mL). As demonstrated here (Fig. 1), if the chosen IVLT is at or above a test’s LOD, that test will be predicted to have near-perfect clinical sensitivity to detect infectious individuals. However, if the true IVLT is below the LOD, clinical sensitivity may be reduced substantially (Fig. 7E). Additionally, when viral loads rise gradually, there is more time between when an individual becomes infectious and when viral loads become detectable by the Ag-RDT. (ii) The second point that the common view misses is the potential for infectious virus in specimen types other than the one tested by the Ag-RDT. We observed presumably infectious viral loads in SA and OPS specimens at all IVLTs from 10^4^ to 10^7^ copies/mL, even while ANS viral loads were well below the Ag-RDT’s LOD. As expected, the Ag-RDT was unable to detect presumably infectious individuals at these time points. In one individual (Fig. 4A), ANS viral loads were undetectable or <10^3^ copies/mL for the first 5 days of infection, resulting in negative Ag-RDT results despite presumably infectious viral loads in SA and OPS specimens. Because nasal Ag-RDTs can only detect individuals with high, presumably infectious viral loads in nasal swabs, individuals with infectious virus in other specimen types are missed (Fig. 7F).

These two points have critical implications for evaluating an Ag-RDT’s ability to detect infectious individuals. Some agent-based outbreak models (5, 54–56) have inferred that low-analytical-sensitivity tests would be effective at mitigating SARS-CoV-2 transmission in a population. Individuals in these simulations are infectious and capable of transmitting infection when viral loads are above a chosen IVLT. These models will overestimate test effectiveness if infectiousness is based only on simulated viral loads in a single tested specimen type and/or if the IVLT chosen is near or above the LOD of the simulated test. Additionally, nearly all studies evaluating Ag-RDT concordance with infectiousness performed viral culture only on a single specimen type (16–19, 35, 36, 38–44), overlooking potentially infectious virus in other types. One of these studies (38) is cited as the basis for CDC (52) recommendations to use repeat ANS Ag-RDTs to improve their clinical sensitivity.

Our third key finding is that use of a combination AN-OP specimen type can significantly improve the performance of Ag-RDTs to detect infectious individuals. Improved detection with an AN-OP combination swab for a different Ag-RDT was recently demonstrated among asymptomatic individuals at a testing center (57). Many countries already authorized and/or implemented the use of combination specimen types for Ag-RDTs, yet this is not the case in the United States, where all at-home Ag-RDTs use nasal swabs.

We acknowledge several study limitations. First, we only evaluated one Ag-RDT. Other Ag-RDTs have different LODs (6, 58); however, equivalence between the clinical sensitivity of this Ag-RDT directly observed versus inferred based on ANS viral loads supports that performance of other Ag-RDTs could also be inferred from quantitative viral load data. Second, we inferred, but did not directly observe, the clinical sensitivity for a combination AN-OP swab. Finally, this study was performed in the context of two SARS-CoV-2 variants (Delta and Omicron) and one geographical area.

Ag-RDTs are useful tools for rapid identification of individuals with high viral loads in the specimen type tested. As discussed above, the utility of Ag-RDTs for detection of infected and presumably infectious individuals is often justified using several assumptions (Fig. 7), in particular that viral loads in all specimen types from an individual at a given time point are similar. Our study demonstrates that this assumption is not justified. Reevaluating assumptions based on new evidence will inform more effective testing strategies, both for SARS-CoV-2 and for other respiratory viral pathogens.

## MATERIALS AND METHODS

### Study design.

We performed a case-ascertained study in the greater Los Angeles County area from November 2021 to March 2022 in which participants prospectively self-collected SA and then ANS and OPS specimens for high-analytical-sensitivity RT-qPCR testing. RT-qPCR testing was performed using the FDA-authorized Zymo Quick SARS-CoV-2 rRT-PCR kit (59), which targets regions of the SARS-CoV-2 N gene and human RNase P gene. RT-qPCR N gene *C_T_* values were used to quantify viral load in the starting specimen, based on a conversion equation generated via a standard curve of known inputs of commercial heat-inactivated SARS-CoV-2 viral particles. Additional details of RT-qPCR testing are provided separately (26). After self-collecting specimens, participants immediately performed an at-home ANS Ag-RDT (Quidel QuickVue At-Home OTC COVID-19 test [49]) per the manufacturer’s instructions. Antigen test results were interpreted by the participant immediately upon completion of the test, and they reported the result and submitted a photograph of the test strip to the research team via a secure REDCap link. Repeat testing of the nasal cavity has been previously shown to maintain diagnostic test performance (60).

RT-qPCR results and viral load quantifications were compared with Ag-RDT results for cross-sectional and longitudinal analyses of Ag-RDT performance. The 228 participants provided 2,107 (ANS), 2,108 (OPS), and 2,114 (SA) time points with valid ANS Ag-RDT and RT-qPCR results for cross-sectional analysis (see supplemental methods). A composite RT-qPCR result was generated for each time point: a participant was considered infected if any of their three specimen types resulted positive by RT-qPCR and uninfected if all specimen types resulted negative by RT-qPCR. Results were inconclusive if at least one specimen type resulted inconclusive while all others resulted negative by RT-qPCR. In total, 2,104 time points had valid, paired ANS Ag-RDT and composite RT-qPCR results. For analyses oriented to early infection, we analyzed longitudinal data from 17 participants who began sampling early in infection (negative in at least one test, RT-qPCR or Ag-RDT, upon enrollment).

All households were infected with either the Delta or Omicron variants (see supplemental methods).

### Statistical analyses.

Positive and negative percent agreement for each specimen type was calculated as the number of specimens with concordant results by RT-qPCR and ANS Ag-RDT over the total number of specimens with positive or negative results, respectively, by RT-qPCR for the given specimen type as reference test.

Quantitative viral loads were used to predict expected results for a specimen tested by a hypothetical assay with a given LOD. Results were also predicted for a computationally contrived AN-OP combination swab, using the higher viral load of the ANS or OPS specimens from a participant at a time point (26). Results were used to calculate inferred positive percent agreement and inferred clinical sensitivity.

Clinical sensitivity was calculated as the number of specimens with either observed or predicted positive results over the total number of infected or infectious time points. We denoted clinical sensitivity as inferred when predicted based on viral load. Error bars indicate 95% confidence intervals calculated as recommended by CLSI (61).

We also presumed that individuals were infectious if viral loads were above the specified infectious viral load threshold (IVLT) of 10^4^, 10^5^, 10^6^, or 10^7^ copies/mL (based on viral culture literature [Table S1]) in at least one specimen type. Differences in the inferred or observed clinical sensitivity from paired RT-qPCR and Ag-RDT data were analyzed using the McNemar exact test using the statsmodels package in Python v3.8.8, with Benjamini-Yekutieli correction.

### Data and materials availability.

The data underlying the results presented in the study can be accessed at CaltechDATA (https://data.caltech.edu/records/20223).
