# Supplementary material for: Daily SARS-CoV-2 Nasal Antigen Tests Miss Infected and Presumably Infectious People Due to Viral Load Differences among Specimen Types
Source: Microbiol Spectr. 2023 Jun 14;11(4):e01295-23. doi: 10.1128/spectrum.01295-23 (PMC10434058; doi:10.1128/spectrum.01295-23)
Supplement: Supplemental file 1 — Supplemental material. Download spectrum.01295-23-s0001.pdf, PDF file, 1.6 MB [file spectrum.01295-23-s0001.pdf]

Supplemental Information for

Daily SARS-CoV-2 Nasal Antigen Tests Miss Infected and Presumably Infectious People Due to Viral-Load Differences Among Specimen Types

Alexander Viloria Winnett[1†], Reid Akana[1†], Natasha Shelby[1†], Hannah Davich[1], Saharai Caldera[1], Taikun Yamada[2,3], John Raymond B. Reyna[2], Anna E. Romano[1], Alyssa M. Carter[1], Mi Kyung Kim[1], Matt Thomson[1], Colten Tognazzini[4], Matthew Feaster[4], Ying-Ying Goh[4], Yap Ching Chew[2,3], Rustem F. Ismagilov[1*]

[1] California Institute of Technology, Pasadena, CA, USA
[2] Pangea Laboratory LLC, Tustin, CA, USA
[3] Zymo Research Corp., Irvine, CA, USA
[4] Pasadena Public Health Department, Pasadena, CA, USA

[†]These authors contributed equally to this report
[*]Corresponding author. 626-395-8130; rustem.admin@caltech.edu

Contents

Supplementary Methods

Supplementary Analyses

Discordance in Participant Interpretation of Antigen Test Results

Faulty Antigen Test Lot

Tables S1-S4

Figures S1-S2

Supplementary References

Detailed Author Contribution Statements

Annotated STARD Checklist

Supplementary Methods

Study Population

This study was approved under Caltech IRB #20-1026. All adult participants provided written informed consent; all minor participants provided verbal assent accompanied by written permission from a legal guardian. Children ages 8-17 years old additionally provided written assent. Eligibility criteria were reported previously[13]; briefly, an individual was eligible to enroll if someone in their home had a recent known exposure or had tested positive for SARS-CoV-2 within the last 5 days. All participants were at least 6 years of age and all participants were fluent in English. Eligibility was determined by the Study Coordinators during a phone interview and/or completion of an online eligibility survey hosted on Qualtrics.

Additional Details about RT-qPCR Testing and Variant Sequencing

Briefly, each day, participants completed an online symptom survey, then self-collected saliva, then anterior-nares swab, then posterior oropharyngeal (throat) swab specimens for RT-qPCR testing. Extraction and RT-qPCR was performed at Pangea Laboratories using the FDA-authorized Quick SARS-CoV-2 RT-qPCR Kit.[30] This assay has a reported LOD of 250 copies/mL of sample, which we also verified prior to study initiation.[25] Details of the quantification of viral load were described previously.[25]

Viral sequencing and variant determination were also performed at Pangea; full methods previously described.[25] Extraction, RT-qPCR, and sequencing operators and supervisors at Pangea Laboratory were blinded to which participant a sample originated from, as well as the infection status and Ag-RDT results of all participants.

Ag-RDT

Participants performed the Ag-RDT according to manufacturer's instructions[1] and reported results and a photograph of their test strip via a secure REDCap server. This Ag-RDT was chosen because it is in use globally,[2] and performance evaluations have been published in several cross-sectional studies.[3-6]

LOD of the Ag-RDT

Conversion from the manufacturer-reported LOD of 1.91×10^4 TCID50/mL of based on commercial heat-inactivated SARS-CoV-2 particles[7] to copies/mL is not possible based on information provided in the FDA documentation for the Quidel QuickVue At-Home OTC COVID-19 Test.[7] Further, the manufacturer was unable to provide this value nor a lot number or certificate of analysis for the heat-inactivated particles. Thus, we were unable to convert this LOD value from TCID50/mL to copies/mL.

Data Used in the Analyses

A total of 2,174 timepoints had a valid, conclusive composite RT-qPCR result, of which 847 timepoints from 90 individuals were classified as infected. Of these 2,174 timepoints, 63 did not have associated Ag-RDT results reported by the participant and 4 had invalid results. Three positive Ag-RDT results were also excluded because they originated from a faulty lot of test strips (see Supplementary Information). A total of 2,107 (nasal swab), 2,108 (throat swab), and 2,114 (saliva) timepoints had valid ANS Ag-RDT and RT-qPCR results (**Fig 2A-F, Fig 3A-C**), and 2,104 timepoints had valid, paired ANS Ag-RDT and composite RT-qPCR results (**Fig 2G-H, Fig 3D-F**).

International Ag-RDT Use Many countries have already authorized and/or implemented the use of combination specimen types for Ag-RDTs, including the United Kingdom, Canada, and Israel.[8-10]

Supplementary Analyses

Confidence intervals were calculated per the guidance in the Clinical Laboratory Standards Institute (CLSI) EPI12 A2 User Protocol for Evaluation of Qualitative Test Performance.[11]

Discordance in Participant Interpretation of Antigen Test Results

Participants interpreted and reported their own antigen test results (positive, negative, or invalid), and photographed their test strips immediately. In the event of an invalid result, study coordinators contacted

participants to request they immediately take an additional test; invalid results were replaced with subsequent valid results, when applicable. Participants recorded their test results and uploaded photos of the test strips to a secure REDCap server immediately after testing. All photographs were inspected by at least two study coordinators blinded to RT-qPCR results. Results as reported by the participants were analyzed and reported here. In 2.5% of antigen tests (56 of 2,153 tests), a pink (positive) test line was visible to two study coordinators in photographs uploaded, but the result was reported as negative by the participant. In most cases the pink lines were faint and may have been overlooked by the participants. It is also possible that in some cases the test was photographed late; per the manufacturer's guidance, the test result is only valid at the 10-min mark. One participant with a dark pink line was queried and reported poor close-range vision; this participant had a housemate help with all further interpretations. In one case from one participant, an invalid result was reported, but a blue control line was visible to two study coordinators. In this manuscript, we used the participants' interpretations in all analyses. Although 2.5% of all rapid antigen test results had discordant interpretations, 14% (33 of 228) of participants had a discordant interpretation; this discordance underlines that user error can substantially affect sensitivity of these at-home tests in real-world settings.

Faulty Antigen Test Lot

In mid-January 2022 we observed that two asymptomatic participants had consecutive positive antigen test results, but negative results by RT-qPCR in all three specimen types tested. Further investigation revealed that the most recently taken false positives from these two participants were from the same antigen strip lot (Quidel QuickVue At-Home OTC COVID-19 Test #152000). A third participant (**Fig 4D**) also had a single false-positive test from this lot the same week. This lot was immediately pulled from circulation in the study, and reported to the manufacturer and to the FDA (via a MedWatch Voluntary Report). Following an IRB amendment, participants began photographing the antigen test strip lot number visible when they reported their Ag-RDT results. Known test results from this faulty lot were marked as invalid and excluded from analysis (**Fig 2**). In one of the 17 participants enrolled during the early period of infection (**Fig 4D**), the antigen test result from this lot is noted with a "?" on his plot, and the datapoint was excluded from subsequent analyses. An investigation of the high rate of false positives was investigated further in a laboratory study using antigen test buffer and commercial nasal fluid from healthy human donors. Full details of that investigation have been reported separately.[12]

In the participant in **Fig 4D**, we continued to observe consistent false-positive Ag-RDTs; with a variety of antigen lots. The participant also tested positive by the Quidel Ag-RDT while testing negative on an iHealth rapid antigen test taken outside of the study on the final day of sampling. This participant tested positive by the Quidel Ag-RDT even >30 days after his first detectable viral load, and when viral load was undetectable by RT-qPCR in all three specimen types. These antigen test strips were not from the lot that yielded consistently false-positive results. The reason for this participant's string of false positives remains unknown.

We observed a negative percent agreement (NPA) of 97% (1,343) antigen negative results of 1,385 ANS RT-qPCR negative results. This is slightly lower than the NPA of 99.2% (95% CI 97.2-99.8%) observed by the Ag-RDT manufacturer.[32] This decrease may be due to the inclusion of additional results from this faulty lot for which we were not able to collect test strip lot information.

Figure software

Fig 4 was created with GraphPad PRISM; **Fig 7** was created using BioRender.

Table S1. Literature providing estimates for infectious viral load thresholds. Relevant literature with paired SARS-CoV-2 viral load and viral culture performed. These studies were reviewed to estimate the lowest viral load at which replication-competent virus was observed, to substantiate possible infectious viral load thresholds (IVLTs). If an exact number was provided in the manuscript, the method is listed as provided, otherwise an approximate value was obtained from review of data shown at the given location in the referenced manuscript. Study Type was listed as Clinical if culture data originated from human clinical specimens; if specimens were collected from humans inoculated with SARS-CoV-2 as part of a research study, the type is listed as Challenge Study. Laboratory study type indicates viral isolates were cultured and subsequently used to compare viral loads at which replication-competent virus was observable. Modeling study type indicates manuscripts without primary culture data that analyzed data from other studies to estimate an infectious viral load. Review indicates a manuscript synthesizing studies that include SARS-CoV-2 viral culture and/or viral load data and does not add new primary data.

**Citation**	**Study Type**	**Minimum Infectious Viral Load (RNA copies/mL)**	**Method**
Stanley S, Hamel Donald J, Wolf Ian D, et al. Limit of Detection for Rapid Antigen Testing of the SARS-CoV-2 Omicron and Delta Variants of Concern Using Live-Virus Culture. J Clin Microbiol 2022; 60(5): e00140-22.	Laboratory	$2.0x10^{2}$	Approximated from Figure 2
Marc A, Kerioui M, Blanquart F, et al. Quantifying the relationship between SARS-CoV-2 viral load and infectiousness. eLife 2021; 10: e69302.	Modeling	$1.0x10^{6}$	Provided (stated as parameter in methods)
Walsh KA, Jordan K, Clyne B, et al. SARS-CoV-2 detection, viral load and infectivity over the course of an infection. *The Journal of infection* 2020; **81**(3): 357-71.	Review	$1.0x10^{5}$	Provided (value provided approximated from primary literature)
van Kampen JJA, van de Vijver DAMC, Fraaij PLA, et al. Duration and key determinants of infectious virus shedding in hospitalized patients with coronavirus disease-2019 (COVID-19). Nature communications 2021; 12(1): 267.	Clinical	$5.0x10^{5}$	Approximated from Figure 1
Perera R, Tso E, Tsang OTY, et al. SARS-CoV-2 Virus Culture and Subgenomic RNA for Respiratory Specimens from Patients with Mild Coronavirus Disease. Emerg Infect Dis 2020; 26(11): 2701-4.	Clinical	$1.0x10^{5}$	Provided
Pickering S, Batra R, Merrick B, et al. Comparative performance of SARS-CoV-2 lateral flow antigen tests and association with detection of infectious virus in clinical specimens: a single-centre laboratory evaluation study. Lancet Microbe 2021; 2(9): e461-e71.	Laboratory	$1.2x10^{6}$	Provided
L'Huillier AG, Torriani G, Pigny F, Kaiser L, Eckerle I. Shedding of infectious SARS-CoV-2 in symptomatic neonates, children and adolescents. *medRxiv* 2020: 2020.04.27.20076778.	Clinical	$1.0x10^{4}$	Approximated from Figure 1
Jones Terry C, Biele G, Mühlemann B, et al. Estimating infectiousness throughout SARS-CoV-2 infection course. Science 2021; 373(6551): eabi5273.	Modeling	$1.0x10^{5}$	Approximated from Figure 2C (based on data from primary literature)
Quicke K, Gallichote E, Sexton N, et al. Longitudinal Surveillance for SARS-CoV-2 RNA Among Asymptomatic Staff in Five Colorado Skilled Nursing Facilities: Epidemiologic, Virologic and Sequence Analysis. *medRxiv* 2020: 2020.06.08.20125989.	Clinical	$1.0x10^{3}$	Approximated from Figure 2B

Puhach O, Adea K, Hulo N, et al. Infectious viral load in unvaccinated and vaccinated patients infected with SARS-CoV-2 WT, Delta and Omicron. *medRxiv* 2022: 2022.01.10.22269010.	Clinical	2.0×10^6	Approximated from Figure 1C
Bal A, Brengel-Pesce K, Gaymard A, et al. Clinical and microbiological assessments of COVID-19 in healthcare workers: a prospective longitudinal study. *medRxiv* 2020: 2020.11.04.20225862.	Clinical	4.5×10^3	Provided in Table S2
Ke R, Martinez PP, Smith RL, et al. Daily longitudinal sampling of SARS-CoV-2 infection reveals substantial heterogeneity in infectiousness. *Nature Microbiology* 2022; **7**(5): 640-52.	Clinical	1.0×10^2	Approximated from Figure 3C/1B and Fig e9
Boucau J, Marino C, Regan J, et al. Duration of Shedding of Culturable Virus in SARS-CoV-2 Omicron (BA.1) Infection. *N Engl J Med* 2022.	Clinical	3.0×10^3	Approximated from Figure 1A
Killingley B, Mann AJ, Kalinova M, et al. Safety, tolerability and viral kinetics during SARS-CoV-2 human challenge in young adults. *Nat Med* 2022; **28**(5): 1031-41.	Challenge Study	2.0×10^2	Approximated from Figure e2B
Wölfel R, Corman VM, Guggemos W, et al. Virological assessment of hospitalized patients with COVID-2019. *Nature* 2020; **581**(7809): 465-9.	Clinical	1.0×10^3	Approximated from Figure 1D
Rhee C, Kanjilal S, Baker M, Klompas M. Duration of Severe Acute Respiratory Syndrome Coronavirus 2 (SARS-CoV-2) Infectivity: When Is It Safe to Discontinue Isolation? *Clin Infect Dis* 2021; **72**(8): 1467-74.	Review	5.0×10^5	Approximated (based on data from primary literature)
Pekosz A, Parvu V, Li M, et al. Antigen-Based Testing but Not Real-Time Polymerase Chain Reaction Correlates With Severe Acute Respiratory Syndrome Coronavirus 2 Viral Culture. *Clin Infect Dis* 2021; **73**(9): e2861-e6.	Clinical	3.2×10^4	Approximated from Figure 1B
Cevik M, Tate M, Lloyd O, Maraolo AE, Schafers J, Ho A. SARS-CoV-2, SARS-CoV, and MERS-CoV viral load dynamics, duration of viral shedding, and infectiousness: a systematic review and meta-analysis. *Lancet Microbe* 2021; **2**(1): e13-e22.	Review	1.0×10^6	Provided (based on data from primary literature)
Berg MG, Zhen W, Lucic D, et al. Development of the RealTime SARS-CoV-2 quantitative Laboratory Developed Test and correlation with viral culture as a measure of infectivity. *J Clin Virol.* 2021;143:104945.	Clinical/Laboratory	1.6×10^4	Provided
Mollan KR, Eron JJ, Krajewski TJ, et al. Infectious Severe Acute Respiratory Syndrome Coronavirus 2 (SARS-CoV-2) Virus in Symptomatic Coronavirus Disease 2019 (COVID-19) Outpatients: Host, Disease, and Viral Correlates. *Clin Infect Dis.* 2022;75(1):e1028-e1036.	Clinical	1.0×10^4	Approximated
La Scola B, Le Bideau M, Andreani J, et al. Viral RNA load as determined by cell culture as a management tool for discharge of SARS-CoV-2 patients from infectious disease wards. *Eur J Clin Microbiol Infect Dis.* 2020;39(6):1059-1061.	Clinical	1.0×10^5	Provided

Table S2. Demographic and Medical Information for Participants Shown in Fig 4. SARS-CoV-2 variant was determined by ANS swab in all cases except individual (B) who had low ANS viral loads so sequencing was from a throat swab. Variant for participant (I) is inferred from the household index case. See also **Table S3**. Some data for participants A-N were reported previously.[13]

Fig 3 panel	Status on enrollment				Months since vaccine			Active Medications	Medical conditions	Gender	Age range (in years)	Race	Ethnicity	SARS-CoV-2 Variant
	Saliva PCR	Throat PCR	Nasal PCR	Nasal antigen	1st dose	2nd dose	3rd dose							
(A)	**neg**	**neg**	**neg**	**neg**	9 [M]	8 [M]	<2 [M]	n/a	n/a	male	40-49	White	not Hispanic	Omicron BA.1.1
(B)	**neg**	**neg**	**neg**	**neg**	11 [JJ]	3 [P]	none	PPI, vitamin/ supplement	obesity, GI condition, anxiety or depression	female	30-39	White	not Hispanic	Omicron BA.1.1
(C)	**inc**	**neg**	**neg**	**neg**	<1 [P]	none	none	acetaminophen	n/a	male	6-11	Multiple Races	not Hispanic	Omicron BA.1.1
(D)	**neg**	**neg**	**neg**	**neg**	10 [M]	9 [M]	2 [M]	none	obesity	male	30-39	Asian or Pacific Islander	not Hispanic	Omicron BA.1.1
(E)	**neg**	**neg**	**neg**	**neg**	>11 [P]	<10 [P]	<3 [P]	allergy medication; acetaminophen, antihistamine, dextromethorphan, phenylephrine HCl, doxylamine	obesity	female	30-39	White	Hispanic	Omicron BA.1
(F)	**neg**	**neg**	**neg**	**neg**	10 [P]	9 [P]	none	vitamin/ supplement	n/a	female	18-29	White	not Hispanic	Omicron BA.1.1
(G)	**neg**	**neg**	**neg**	**neg**	<2 [P]	<1 [P]	none	vitamin/ supplement	n/a	male	6-11	White	not Hispanic	Omicron BA.1.1
(H)	**neg**	**neg**	**neg**	**neg**	10 [M]	9 [M]	2 [M]	vitamin/ supplement	n/a	female	40-49	White	not Hispanic	Omicron BA.1.1
(I)	**neg**	**neg**	**neg**	**neg**	10 [P]	9 [P]	none	antibiotic, vitamin/ supplement	obesity	male	18-29	White	Hispanic	Omicron BA.1.1 (index case)
(J)	*pos*	*pos*	**inc**	**neg**	9 [M]	8 [M]	<2 [M]	vitamin/ supplement	anxiety or depression	female	40-49	White	not Hispanic	Omicron BA.1.1
(K)	*pos*	*pos*	**inc**	**neg**	9.5 [M]	8.5 [M]	0.5 [P]	NSAID	n/a	male	40-49	White	not Hispanic	Omicron BA.1.1
(L)	*pos*	*pos*	*pos*	**neg**	11 [P]	10 [P]	2 [P]	allergy medication, diabetes medication, cholesterol medication	diabetes, high blood pressure, obesity, asthma, sleep apnea, GI condition	female	50-59	Multiple Races	not Hispanic	Omicron BA.1.1
(M)	*pos*	*pos*	**neg**	**neg**	10 [M]	9 [M]	2 [M]	SSRI	oveweight, anxiety or depression	male	50-59	White	not Hispanic	Omicron BA.1.1
(N)	*pos*	**neg**	*pos*	**neg**	5 [P]	4[P]	none	none	n/a	female	12-17	White	not Hispanic	Omicron BA.1.1
(O)	*pos*	*pos*	*pos*	**neg**	10 [P]	9 [P]	1 [P]	vitamin/ supplement	anxiety or depression	female	40-49	White	not Hispanic	Omicron BA.1.1
(P)	*pos*	*pos*	*pos*	**neg**	13 [P]	12 [P]	3.5 [P]	none	n/a	male	18-29	Asian	not Hispanic	Omicron BA.1.1
(Q)	*pos*	*pos*	*pos*	**neg**	9[P]	8[P]	<0.5 [P]	acetaminophen, antihistamine, dextromethorphan, phenylephrine HCl, doxylamine	obesity	female	12-17	White	Hispanic	Omicron BA.1

* Months from vaccine date are given relative to enrollment date
# Vaccine abbreviations: [P], Pfizer-BioNTech COVID-19 Vaccine (COMIRNATY); [M], Moderna COVID-19 Vaccine (Spikevax); [JJ], Johnson & Johnson
NQ, not quantifiable; viral load was below the test LOD (250 SARS-CoV-2 RNA copies/mL)
** Participants were asked to report both sex at birth and current gender identity; all participants in this cohort responded cis-gender identities to sex at birth

Table S3. Demographics of the 17-participant cohort shown in Fig 4. Additional detailed information on each participant can be found in Table S2.

**Sex***		
Male	8	47.1%
Female	9	52.9%
**Age**		
6-11	2	11.8%
12-17	2	11.8%
18-29	3	17.6%
30-39	3	17.6%
40-49	5	29.4%
50-59	2	11.8%
**Race**		
White	13	76.5%
Asian or Pacific Islander	2	11.8%
Multiple Races	2	11.8%
**Ethnicity**		
Hispanic	3	17.6%
Non-Hispanic	14	82.4%
**Tobacco Smoker or Vape User History**		
Current	0	0.0%
Former	2	11.8%
Never	15	88.2%
**Active Medications and Supplements**		
Vitamins/Supplements	7	41.2%
Acetaminophen/NSAIDs	4	23.5%
Allergy medications/Antihistamines	3	17.6%
Antibiotics/Antivirals	1	5.9%
**Medical Comorbidities**		
Asthma	1	5.9%
Anxiety or Depression	3	17.6%
Diabetes	1	5.9%
Overweight/Obesity	7	41.2%
GI condition	2	11.8%
**SARS-CoV-2 Vaccination Status**		
Partially Vaccinated	1	5.9%
Completed Vaccination	5	29.4%
Fully vaccinated and boosted	11	64.7%
No SARS-CoV-2 vaccines reported	0	0.0%

*Participants were asked to report both sex at birth and current gender identity; all participants in this cohort responded cis-gender identities to sex at birth

	Specimen Type	LOD (copies/mL)		Specimen Type	LOD (copies/mL)	Infectious Viral Load Threshold (copies/mL)			
						10^4	10^5	10^6	10^7
Infectious Period	ANS Ag-RDT	Observed	vs	ANS Inferred	10^6	47.5 vs 44.9%	54.8 vs 53.2	63.3 vs 64.3	63.0 vs 64.8
	ANS Ag-RDT	Observed	vs	OPS Inferred	10^6	47.5 vs 28.1%	54.8 vs 33.3	63.3 vs 40.3	63.0 vs 40.6
	ANS Ag-RDT	Observed	vs	AN-OP Inferred	10^6	47.5 vs 57.3%	54.8 vs 68.0	63.3 vs 82.2	63.0 vs 82.8
	AN-OP Inferred	10^6	vs	ANS Inferred	10^6	57.3 vs 44.9%	68.0 vs 53.2	82.2 vs 64.3	82.8 vs 64.8
	AN-OP Inferred	10^6	vs	OPS Inferred	10^6	57.3 vs 28.1%	68.0 vs 33.3	82.2 vs 40.3	82.8 vs 40.6

Table S4. Comparisons of the Observed and Inferred Performance of Low-Analytical-Sensitivity Diagnostic Tests (Ag-RDTs) to Detect Presumed Infectious Individuals. Individuals were presumed infectious for the period between first specimen (of any type) with a viral load above the infectious viral load threshold (10^4, 10^5, 10^6, or 10^7 copies/mL) until all specimen types were below the IVLT. -Comparison of the clinical sensitivities to detect infectiousness at IVLTs of 10^4 to 10^7 across specimen types was performed using the McNemar Exact Test, for given comparisons across specimen type. ANS Ag-RDT vs ANS with LOD 10^6 copies/mL was tested using a two-tailed McNemar Exact Test; all other combinations use a one-tailed McNemar exact test. *P*-values were adjusted using a Benjamini–Yekutieli correction to account for multiple hypotheses being tested. Comparisons resulting in *p*-values <0.05 were considered statistically significant, and are indicated in red. SA, saliva; ANS, anterior-nares swab; OPS, oropharyngeal swab; AN–OP, anterior-nares–oropharyngeal combination swab; LOD, limit of detection.

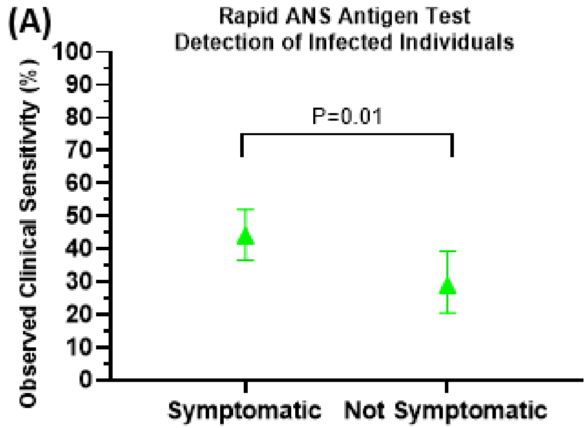

Figure S1. Relationship Between Symptoms and Viral Load. The observed clinical sensitivity of the rapid antigen test to detect infection is plotted for timepoints when the cohort of 17 participants enrolled early in the course of the infection either reported at least one symptom (Symptomatic) or did not report any symptoms (Not Symptomatic). An upper-tailed Fished exact test was performed to determine whether Ag-RDT performance at symptomatic timepoints was significantly higher than timepoints when participants experienced no symptoms.

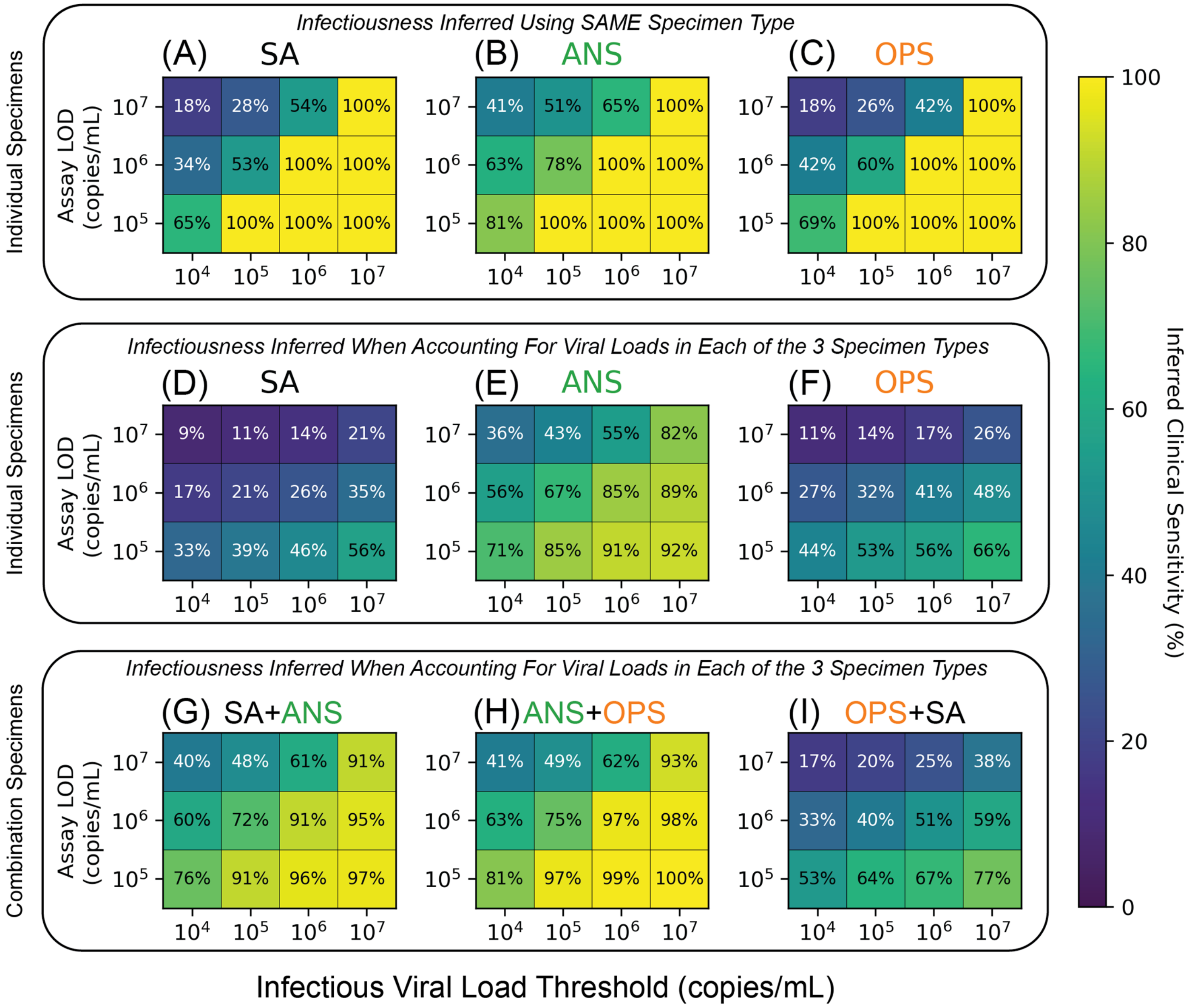

Figure S2. Effect of Test LOD and Infectious Viral-Load Threshold (IVLT) on Inferred Clinical Sensitivity of Contrived Specimen Combinations. Clinical sensitivities of assays with varying LOD and IVLT for single specimen types (A-F) and contrived combination specimen types (G-I). Samples were deemed infectious if its own viral load surpassed the IVLT (A-C), or if the viral load any sample collected from the same individual at the same timepoint surpassed the IVLT (D-I). Contrived combination specimens (G-I) were calculated by taking the max viral load over the two specified specimen types.

Supplementary References

1. Quidel. Quidel QuickVue At-Home OTC COVID-19 Test - User Instructions. 2021. https://quickvueathome.com/wp-content/uploads/2021/05/EF1479701EN00_QV_At-Home_Web_Instructions_English_052421.pdf.

2. PATH. Global Availability of COVID-19 Diagnostic Tests. 2022. https://www.path.org/programs/diagnostics/covid-dashboard-global-availability-covid-19-diagnostic-tests/.

3. Kepczynski CM, Genigeski JA, Koski RR, Bernknopf AC, Konieczny AM, Klepser ME. A systematic review comparing at-home diagnostic tests for SARS-CoV-2: Key points for pharmacy practice, including regulatory information. *J Am Pharm Assoc (2003)* 2021; **61**(6): 666-77.e2.

4. Hardick J, Gallagher N, Sachithanandham J, et al. Evaluation of Four Point of Care (POC) Antigen Assays for the Detection of the SARS-CoV-2 Variant Omicron. *Microbiol Spectr* 2022: e0102522.

5. Bourassa L, Perchetti GA, Phung Q, et al. A SARS-CoV-2 Nucleocapsid Variant that Affects Antigen Test Performance. *J Clin Virol* 2021; **141**: 104900.

6. Tinker SC, Prince-Guerra JL, Vermandere K, et al. Evaluation of self-administered antigen testing in a college setting. *Virol J* 2022; **19**(1): 202.

7. FDA. Quidel QuickVue At-Home OTC COVID-19 Test - Instructions for Use (IFU). 2021. https://www.fda.gov/media/147265/download

8. National Health Service. How to use an NHS rapid lateral flow test for coronavirus (COVID-19). 2021. https://www.nhs.uk/conditions/coronavirus-covid-19/testing/how-to-do-a-test-at-home-or-at-a-test-site/how-to-do-a-rapid-lateral-flow-test/.

9. Ontario Health Canada. COVID-19 Rapid Antigen Tests: How to Collect a Sample. 2022. https://www.ontariohealth.ca/sites/ontariohealth/files/2022-02/COVID-19RapidAntigenTests-HowtoCollectaSample.pdf.

10. Reuters. Israel's Health Ministry recommends throat swab for COVID-19 tests while U.S. FDA warns against. 2022. https://www.ctvnews.ca/health/coronavirus/israel-s-health-ministry-recommends-throat-swab-for-covid-19-tests-while-u-s-fda-warns-against-1.5734130.

11. CLSI. EP12-A2 User Protocol for Evaluation of Qualitative Test Performance, 2nd edition; 2008.

12. Carter Alyssa M, Viloria Winnett A, Romano Anna E, Akana R, Shelby N, Ismagilov RF. Laboratory Evaluation Links Some False-Positive COVID-19 Antigen Test Results Observed in a Field Study to a Specific Lot of Test Strips. *Open Forum Infectious Diseases* 2023: 10.1093/ofid/ofac701/6972331.

13. Viloria Winnett A, Akana R, Shelby N, et al. Extreme differences in SARS-CoV-2 viral loads among respiratory specimen types during presumed pre-infectious and infectious periods. *PNAS Nexus.* doi: 10.1093/pnasnexus/pgad033

AUTHOR CONTRIBUTIONS (listed alphabetically by last name):

Reid Akana (RA): Collaborated with AVW in creating digital participant symptom surveys; assisted with data quality control/curation with NS, HD, SC; created current laboratory information management system (LIMS) for specimen logging and tracking. Creation of iOS application for sample logging/tracking. Configured an SQL database for data storage. Created an Apache server and websites to view study data. Configured FTPS server to catalog PCR data. Wrote a Python package to access study data. Trained study coordinators on SQL. Troubleshooting and QC of LIMS. Made Figures 3, 5, S2, S3, S4, S5. Wrote and edited the manuscript with AVW and NS.

Alyssa M. Carter (AMC): Assisted with the inventory and archiving of >6,000 samples at Caltech; coordinated shipment of samples to Caltech with AER and JRBR; assisted with procurement of antigen tests; assisted with organizing volunteers and making participant kits; assisted AER in developing and implementing QC for participant kits. Led the in-lab investigation of antigen false-positive results; designed and performed experiments for lot analysis of the Quidel QuickVue At-Home Covid-19 tests. Provided feedback and edited the manuscript.

Yap Ching Chew (YCC): Primary liaison with Caltech team. Prepared and provided Zymo SafeCollect kits and related materials to Caltech team. Supervised the extraction, PCR, and QC teams at Pangea Laboratory. Sent PCR results daily to Caltech team. Arranged for Pangea team to perform viral-variant sequencing on selected samples; reported results and provided sequencing files.

Saharai Caldera (SC): Study coordinator; recruited, enrolled and maintained study participants with NS and HD; study-data quality control, curation and archiving with RA, NS, HD and MKK; supplies acquisition with AER, NS, HD and MKK.

Hannah Davich (HD): Lead study coordinator; co-wrote participant informational sheets with NS; developed recruitment strategies and did outreach with NS; participant kit creation and co-coordinated kit-making by volunteers with AER; recruited, enrolled and maintained study participants with NS and SC; managed the study-coordinator inventory; study-data quality control, curation and archiving with RA, NS, SC and MKK; supplies acquisition with AER, NS, SC and MKK.

Matthew Feaster (MF): Co-investigator; collaborated with AVW, MMC, NS, YG, RFI on study design and recruitment strategies; provided guidance and expertise on SARS-CoV-2 epidemiology and local trends.

Ying-Ying Goh (Y-YG): Co-investigator; collaborated with AVW, MMC, NS, MF, RFI on study design and recruitment strategies; provided guidance and expertise on SARS-CoV-2 epidemiology and local trends.

Rustem F. Ismagilov (RFI): Principal investigator; collaborated with AVW, MMC, NS, MF, YYG on study design and recruitment strategies; provided leadership, technical guidance, and oversight of all analyses; was responsible for obtaining the primary funding for the study.

Mi Kyung Kim (MKK): Study coordinator (part-time); maintaining participants with NS, HD, and SC; study-data quality control, curation and archiving with RA, NS, SC and HD; supplies acquisition with AER, NS, SC and HD; collected contact info for local health centers for recruitment outreach; assembled Table S2 with NS.

John Raymond B. Reyna (JRBR): Organized sample labeling and short-term storage of all samples at Pangea Laboratories. Arranged shipment of all samples to Caltech team. Assisted with processing of the specimens.

Anna E. Romano (AER): Co-coordinated kit-making by volunteers with HD; implemented QC process for kit-making; participated in kit making; managed logistics for the inventory and archiving of >6,000 samples at Caltech; supplies acquisition with HD, NS, SC and MKK; assisted with securing funding; compiled antigen lot data to assist false-positive antigen test investigation; organized and performed QC on sequencing data. Provided feedback and edited the manuscript.

Natasha Shelby (NS): Study administrator; collaborated with AVW, RFI, YG, MF on initial study design and recruitment strategies; co-wrote IRB protocol and informed consent with AVW; co-wrote enrollment

questionnaire and post-study questionnaire with AVW; initiated the collaboration with Zymo and served as primary liaison throughout study; reviewed pilot sampling data and amended instructional sheets/graphics for specimen collections in collaboration with Zymo; co-wrote participant informational sheets with HD; hired, trained, and supervised the study-coordinator team; developed recruitment strategies and did outreach with HD; recruited, enrolled and maintained study participants with HD and SC; co-developed participant keep/drop criteria with AVW; performed the daily upload, review, and QC of PCR data received from Zymo; made the daily participant keep/drop decisions based on viral-load results and trajectories in each household; made all phone calls to alert presumptive positives of their status and provide resources; study-data quality control, curation and archiving with RA, HD, SC and MKK; archiving of all participant data and antigen-test photographs; supplies acquisition with AER, HD, SC and MKK; assisted with securing funding; managed the overall study budget; assembled Fig 1 with AVW; assembled Table S2 with MKK; assembled Table S3; created Fig 4 with AVW; managed citations and reference library; verified the underlying data with AVW and RA; co-wrote and edited the manuscript with AVW and RA.

Matt Thomson (MT): Assisted with statistical approach and analyses.

Colten Tognazzini (CT): Coordinated the recruitment efforts at PPHD with case investigators and contact tracers; provided guidance and expertise on SARS-CoV-2 epidemiology and local trends.

Alexander Viloria Winnett (AVW): Collaborated with NS, RFI, YG, MF on initial study design and recruitment strategies; co-wrote IRB protocol and informed consent with NS; co-wrote enrollment questionnaire and post-study questionnaire with NS; co-developed participant keep/drop criteria with NS; funding acquisition; designed and coordinated LOD validation experiments; selected and prepared specimen for viral-variant sequencing with NS, YC, and AER; assisted with the inventory and archiving of >6,000 specimen at Caltech with AER and AMC; minor role supporting outreach by HD and NS; minor role supporting kit-making by AER, HD and AMC; verified the underlying data with NS and RA; major contributor to reference organization and selection; assembled Fig 1 with NS; created Fig 4 with NS; performed analysis and prepared Fig 2, Fig 6, Fig 7, Fig S1, Table S1, and Table S4. Co-wrote and edited the manuscript with NS and RA.

Taikun Yamada (TY): Performed the RT-qPCR COVID-19 testing at Pangea Laboratory.

Section & Topic	No	Item
**TITLE OR ABSTRACT**		
	**1**	Identification as a study of diagnostic accuracy using at least one measure of accuracy (such as sensitivity, specificity, predictive values, or AUC)
**ABSTRACT**		
	**2**	Structured summary of study design, methods, results, and conclusions (for specific guidance, see STARD for Abstracts)
**INTRODUCTION**		
	**3**	Scientific and clinical background, including the intended use and clinical role of the index test
	**4**	Study objectives and hypotheses
**METHODS**		
Study design	**5**	Whether data collection was planned before the index test and reference standard were performed (prospective study) or after (retrospective study)
Participants	**6**	Eligibility criteria
	**7**	On what basis potentially eligible participants were identified (such as symptoms, results from previous tests, inclusion in registry)
	**8**	Where and when potentially eligible participants were identified (setting, location and dates)
	**9**	Whether participants formed a consecutive, random or convenience series
Test methods	**10a**	Index test, in sufficient detail to allow replication
	**10b**	Reference standard, in sufficient detail to allow replication
	**11**	Rationale for choosing the reference standard (if alternatives exist)
	**12a**	Definition of and rationale for test positivity cut-offs or result categories of the index test, distinguishing pre-specified from exploratory
	**12b**	Definition of and rationale for test positivity cut-offs or result categories of the reference standard, distinguishing pre-specified from exploratory
	**13a**	Whether clinical information and reference standard results were available to the performers/readers of the index test
	**13b**	Whether clinical information and index test results were available to the assessors of the reference standard
Analysis	**14**	Methods for estimating or comparing measures of diagnostic accuracy
	**15**	How indeterminate index test or reference standard results were handled
	**16**	How missing data on the index test and reference standard were handled
	**17**	Any analyses of variability in diagnostic accuracy, distinguishing pre-specified from exploratory
	**18**	Intended sample size and how it was determined
**RESULTS**		
Participants	**19**	Flow of participants, using a diagram
	**20**	Baseline demographic and clinical characteristics of participants
	**21a**	Distribution of severity of disease in those with the target condition
	**21b**	Distribution of alternative diagnoses in those without the target condition
	**22**	Time interval and any clinical interventions between index test and reference standard
Test results	**23**	Cross tabulation of the index test results (or their distribution) by the results of the reference standard
	**24**	Estimates of diagnostic accuracy and their precision (such as 95% confidence intervals)
	**25**	Any adverse events from performing the index test or the reference standard
**DISCUSSION**		
	**26**	Study limitations, including sources of potential bias, statistical uncertainty, and generalisability
	**27**	Implications for practice, including the intended use and clinical role of the index test
**OTHER INFORMATION**		
	**28**	Registration number and name of registry
	**29**	Where the full study protocol can be accessed
	**30**	Sources of funding and other support; role of funders

CC BY SA

STARD 2015

AIM

STARD stands for "Standards for Reporting Diagnostic accuracy studies". This list of items was developed to contribute to the completeness and transparency of reporting of diagnostic accuracy studies. Authors can use the list to write informative study reports. Editors and peer-reviewers can use it to evaluate whether the information has been included in manuscripts submitted for publication.

EXPLANATION

A **diagnostic accuracy study** evaluates the ability of one or more medical tests to correctly classify study participants as having a **target condition.** This can be a disease, a disease stage, response or benefit from therapy, or an event or condition in the future. A medical test can be an imaging procedure, a laboratory test, elements from history and physical examination, a combination of these, or any other method for collecting information about the current health status of a patient.

The test whose accuracy is evaluated is called **index test.** A study can evaluate the accuracy of one or more index tests. Evaluating the ability of a medical test to correctly classify patients is typically done by comparing the distribution of the index test results with those of the **reference standard**. The reference standard is the best available method for establishing the presence or absence of the target condition. An accuracy study can rely on one or more reference standards.

If test results are categorized as either positive or negative, the cross tabulation of the index test results against those of the reference standard can be used to estimate the **sensitivity** of the index test (the proportion of participants *with* the target condition who have a positive index test), and its **specificity** (the proportion *without* the target condition who have a negative index test). From this cross tabulation (sometimes referred to as the contingency or "2x2" table), several other accuracy statistics can be estimated, such as the positive and negative **predictive values** of the test. Confidence intervals around estimates of accuracy can then be calculated to quantify the statistical **precision** of the measurements.

If the index test results can take more than two values, categorization of test results as positive or negative requires a **test positivity cut-off**. When multiple such cut-offs can be defined, authors can report a receiver operating characteristic (ROC) curve which graphically represents the combination of sensitivity and specificity for each possible test positivity cut-off. The **area under the ROC curve** informs in a single numerical value about the overall diagnostic accuracy of the index test.

The **intended use** of a medical test can be diagnosis, screening, staging, monitoring, surveillance, prediction or prognosis. The **clinical role** of a test explains its position relative to existing tests in the clinical pathway. A replacement test, for example, replaces an existing test. A triage test is used before an existing test; an add-on test is used after an existing test.

Besides diagnostic accuracy, several other outcomes and statistics may be relevant in the evaluation of medical tests. Medical tests can also be used to classify patients for purposes other than diagnosis, such as staging or prognosis. The STARD list was not explicitly developed for these other outcomes, statistics, and study types, although most STARD items would still apply.

DEVELOPMENT

This STARD list was released in 2015. The 30 items were identified by an international expert group of methodologists, researchers, and editors. The guiding principle in the development of STARD was to select items that, when reported, would help readers to judge the potential for bias in the study, to appraise the applicability of the study findings and the validity of conclusions and recommendations. The list represents an update of the first version, which was published in 2003.

More information can be found on http://www.equator-network.org/reporting-guidelines/stard.

CC BY SA
